# Selective Acetogenins and Their Potential as Anticancer Agents

**DOI:** 10.3389/fphar.2019.00783

**Published:** 2019-07-18

**Authors:** Nadia Jacobo-Herrera, Carlos Pérez-Plasencia, Víctor Alberto Castro-Torres, Mariano Martínez-Vázquez, Alma Rosa González-Esquinca, Alejandro Zentella-Dehesa

**Affiliations:** ^1^Unidad de Bioquímica, Instituto Nacional de Ciencias Médicas y Nutrición Salvador Zubiran, Ciudad de México, Mexico; ^2^Laboratorio de Genómica Funcional, Unidad de Biomedicina, Facultad de Estudios Superiores (FES) Iztacala, Universidad Nacional Autónoma de México (UNAM), Tlalnepantla, Mexico; ^3^Laboratorio de Genómica, Instituto Nacional de Cancerología, Ciudad de México, Mexico; ^4^Posgrado en Ciencias Biomédicas, Universidad Nacional Autónoma de México, Ciudad de México, Mexico; ^5^Departamento de Productos Naturales, Instituto de Química, Universidad Nacional Autónoma de México, Ciudad de México, Mexico; ^6^Laboratorio de Fisiología y Química Vegetal, Instituto de Ciencias Biológicas, Universidad de Ciencias y Artes de Chiapas, Tuxtla Gutiérrez, Mexico; ^7^Departamento de Medicina Genómica y Toxicología Ambiental & Programa Institucional de Cáncer de Mama, Instituto de Investigaciones Biomédicas, Universidad Nacional Autónoma de México, Ciudad de México, Mexico

**Keywords:** acetogenins, cytotoxicity, apoptosis, cell death, cell cycle, autophagy

## Abstract

The Kingdom Plantae has provided several successful drugs for the treatment of different diseases, including cancer, and continues to be a source of new possible therapeutic molecules. For example, the annonaceous acetogenins (AAs) are secondary metabolites found in the Annonaceae family, which are plants employed in traditional medicine for the treatment of cancer and various other diseases. These polyketides are inhibitors of Complex I in the respiratory chain of tumor cells, a process that is closely related to tumor metabolism, cell death, apoptosis, and autophagy. The goal of this review is to update readers on the role of the AAs as antitumor agents using *in vitro* and *in vivo* studies to demonstrate their importance in the area of oncology drug discovery. For this purpose, we performed a literature search in the PubMed scientific database using a range of keywords, including acetogenins and cancer, acetogenins antitumor activity, acetogenins and cytotoxicity, and acetogenins mechanism of action, among others. As a result, we found that the AAs are cytotoxic compounds that can induce apoptosis, cell cycle arrest, and autophagy *in vitro*, in addition to exhibiting tumor growth inhibition *in vivo*. The functional group related to their antineoplastic activity is suggested to be the mono or bis tetrahydrofuran ring accompanied by two or more hydroxy groups. The versatility of the AA bioactivity therefore renders them potential therapeutic agents for cancer treatment. It is therefore apparent that nature is worth further examination to aid in the discovery of more effective, accurate, and less harmful therapies in the fight against cancer.

## Introduction

The use of natural remedies is an ancient tradition still in practice, with >60% of the world’s population addressing various health issues, including cancer, using traditional medicine as a first choice (Sultana et al., 2014). However, cancer treatment is complex, and current possibilities for patients depend on the cancer type and stage, in addition to the age, sex, and overall health of the patient. Although chemotherapy is usually successful during the early stages of cancer, its efficacy depends on the drug administration scheme and the physiological condition of the patient. Nevertheless, the main concern regarding chemotherapy is its toxicity, as the drugs employed tend to affect cancer cells as well as normal cells, with a high proliferative index generating collateral damage in patients. Moreover, cancer cells can develop treatment resistance, exhibiting metastasis, and thereby lessening the response to treatment and reducing the possibility of disease-free survival. Therefore, one of the key challenges in drug discovery is demising the toxicity of chemotherapeutic agents and developing more effective and efficient drugs to improve treatments, recovery times, and overall patient quality of life.

Natural products have provided a rich source of chemical structures for the development of anti-cancer treatments. In the area of oncology pharmaceutics, 49% of the drugs employed in chemotherapy are derived from or inspired by natural sources such as plants, microorganisms, and marine organisms (Newman and Cragg, 2016). Examples include vinca alkaloids and taxanes (tubulin-binding agents), in addition to podophyllotoxins, anthracyclines, and etoposides (topoisomerase inhibitors) (Da Rocha et al., 2001; Nobili et al., 2009). These examples illustrate the potential of natural products in drug discovery.

**Figure 1:**
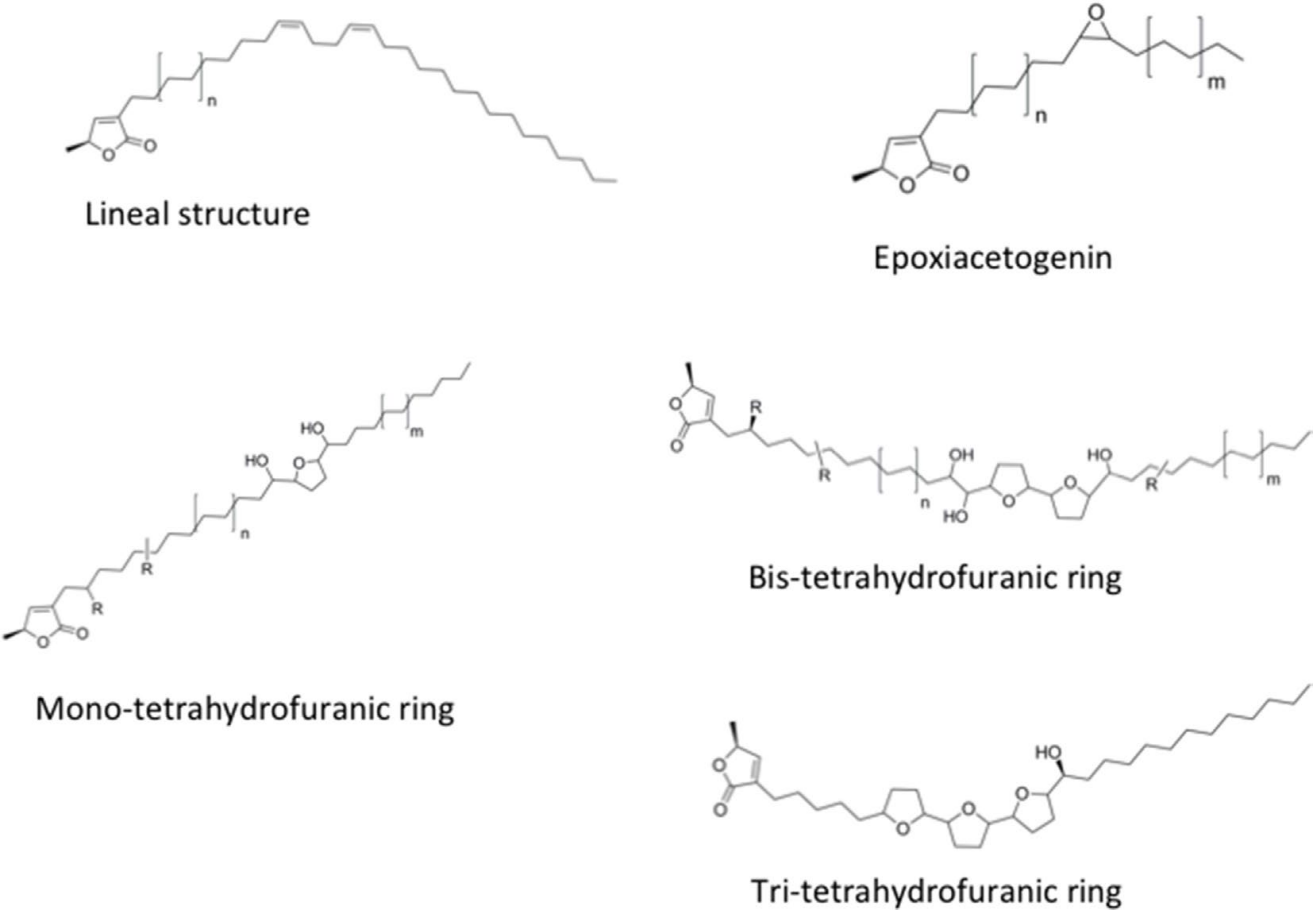
Acetogenins nucleus: lineal, epoxic, mono-tetrahydrofuran (THF), bis-THF, and tris-THF.

The AAs are secondary metabolites produced by the Annonaceae family (**Figure 1**). In traditional medicine, the fruit of the *Annona* genus is used for the treatment of fever, pain, rheumatism, diarrhea, and arthritis, and its leaves for diabetes, headaches, and insomnia (Moghadamtousi et al., 2015a). *Annona reticulate* has been employed in Africa as an anti-dysenteric and anti-helminthic treatment, and *A. squamosa* is used in India for the treatment of various conditions including malignant tumors (Savithramma et al., 2011). Furthermore, *A. muricata* is a popular medicinal remedy in America, Africa, and India for the treatment of cancer (Moghadamtousi et al., 2015a), while in Mexico, *A. macroprophyllata*, *A. muricata*, and *A. purpurea* are used for the treatment of skin tumors and gastric cancer (Alonso-Castro et al., 2011; Brindis et al., 2013). According to an ethnopharmacological review of medicinal plants in Mexico, the genus *Annona* could also be explored for the treatment of colon cancer (Jacobo-Herrera et al., 2016). This evidence indicates the AAs are molecules with significant bioactive potential.

Over 500 AAs have been described to date; these compounds are characterized by a long aliphatic chain with an α, β-unsaturated γ-lactone ring and 0–3 tetrahydrofuran (THF) rings (Yuan et al., 2015), and have shown cytotoxic activity against different human cancer cell lines (i.e., lung, breast, colon, kidney, pancreas, prostate, liver, and bladder) (reviewed in Zeng et al., 1996; Chávez and Mata, 1998; Wang et al., 2002; Yuan et al., 2003; Mangal et al., 2016; Miao et al., 2016a; Miao et al., 2016b) besides *in vivo* antitumor activity (Chen et al., 2012a; Chen et al., 2012b). Herein, we reviewed the most recent reports about AAs in the drug discovery–oncology field.

## Materials and Methods

We performed a search in the PubMed database using the following keyword terms: “acetogenins and cancer,” “acetogenins antitumor activity,” “acetogenins cytotoxicity,” and “acetogenins mechanism of action,” among other combinations. We reviewed articles published over the last 15 years that were based on two criteria: plants with medicinal use and the bioassay-guided fractionation methodology. The *in vitro* data reported herein are based exclusively on the activity of pure compounds, while for *in vivo* studies, herbal extracts were also considered due to the lack of assays in animal models.

## Results and Discussion

### *In Vitro* Studies

In the field of natural products, the search for bioactive molecules tends to involve a bioassay-guided fractionation of extracts to find the *master* molecule or molecules responsible for the activity. However, many secondary metabolites can target multiple hallmarks of cancer. In general, the pharmaceutical industry is interested in drugs that exhibit more than one molecular interaction and encompass various molecular targets.

Acetogenins are molecules with great potential for future cancer therapy. Their most prominent biologic activity is inhibition of the mitochondrial Complex I due to their bis-THF structure. Indeed, it was previously reported that the mono-THF AAs bearing an alkyl chain that links the lactone moiety with the THF group are noncompetitive inhibitors of Complex I (i.e., NADH: ubiquinone oxidoreductase) in the respiratory chain, which leads to a blockade of phosphorylative oxidation and a subsequent decrease in ATP production (Tormo et al., 1999; Chen et al., 2012). Such inhibition involves a large group of pathways that can induce cell death, including apoptosis and autophagy, or act in other metabolic networks as inhibitors of the lactate dehydrogenase A enzyme, as an antioxidant, or by arresting the cell cycle.

Moreover, *A. muricata* (Moghadamtousi et al., 2015a) and *A. squamosa* L. (Chen et al., 2012a; Chen et al., 2012b; Chen et al., 2012c; Chen et al., 2016) have been reported to have cytotoxic activity against several human or other mammal cancer cell lines. Particularly, the AAs annosquatins A (**1**) and B (**2**) exhibit selectivity towards the MCF-7 and A-549 cell lines, respectively (Chen et al., 2012c). **Table 1** shows the most recently discovered AAs exhibiting cytotoxic activity, in addition to other less recent compounds that exhibit antineoplastic activity *in vivo* (**Table 2**).

**Table 1:** Cytotoxic activity of acetogenins *in vitro* in different cancer cell lines.

Plant species	Acetogenin	Cytotoxicity/cancer cell line	Functional groups	References
*Annona cornifolia* A. St.-Hil	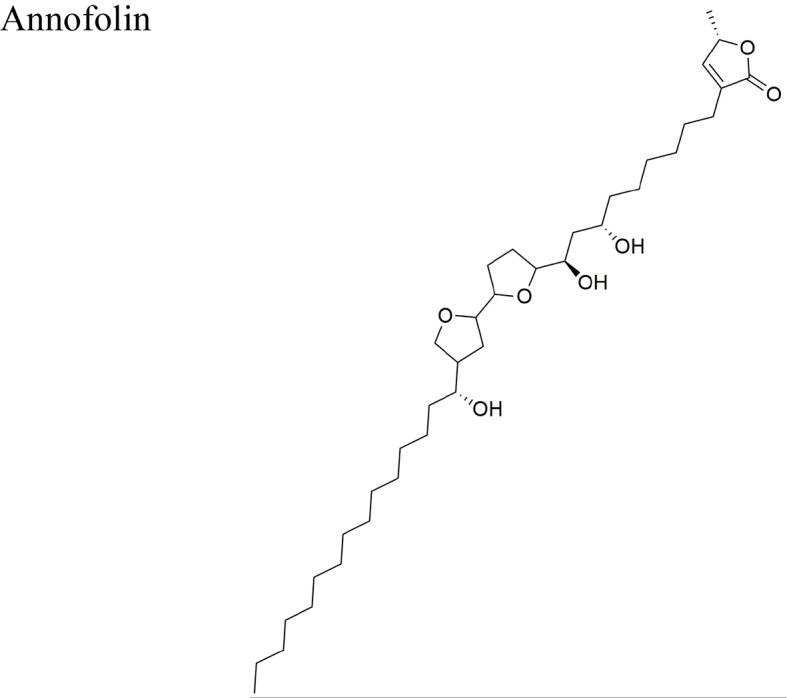	Acetogenins isolated from *A. cornifolia*: squamocin M, annofolin, isolongimicin B, glaucanisin, and annotacin IC_50_ in the range of <3.0 × 10^−1^ to <3.2 × 10^−1^ µM/MCF-7	Annofolin the most active acetogenin of the group from *A. cornifolia* (IC_50_ = < 3.0 × 10^1^) is a C-37 acetogenin with 9 carbon atoms between the OH-flanked THF and the γ-lactone and a stereochemical arrangement of *erythro/trans/threo/* *trans/threo* around THF rings	Lima et al., 2014
*Annona diversifolia* Saff.	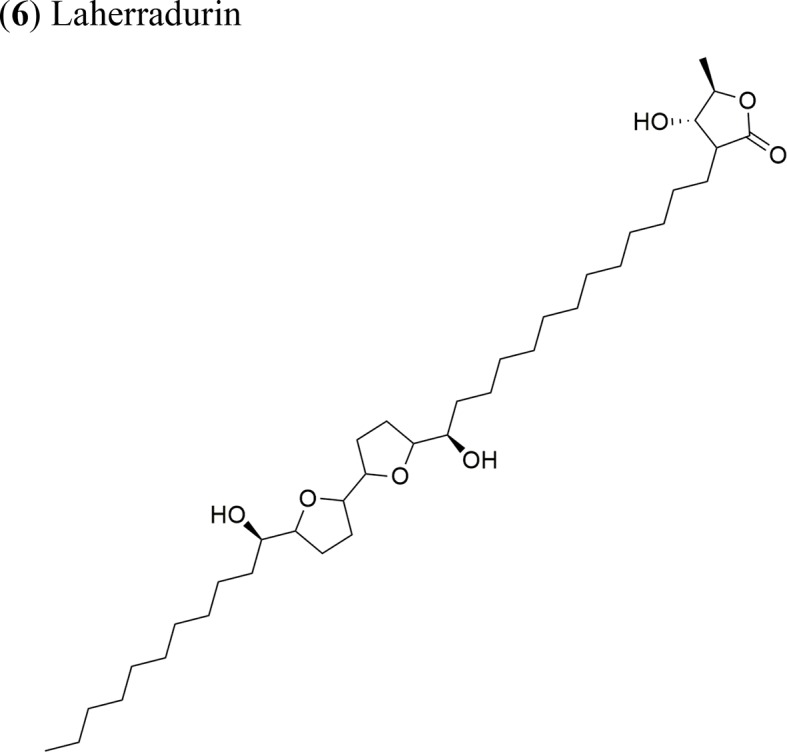	Laherradurin: ED_50_ = 0.015 µg/ml/HeLa and SW480 Cherimolin-2: ED_50_ = 0.05 µg/ml/HeLa and SW480	Adjacent bis THF with β-hydroxy γ-methyl γ-lactone Non-adjacent bis THF γ-lactone	Schlie-Guzmán et al., 2009
	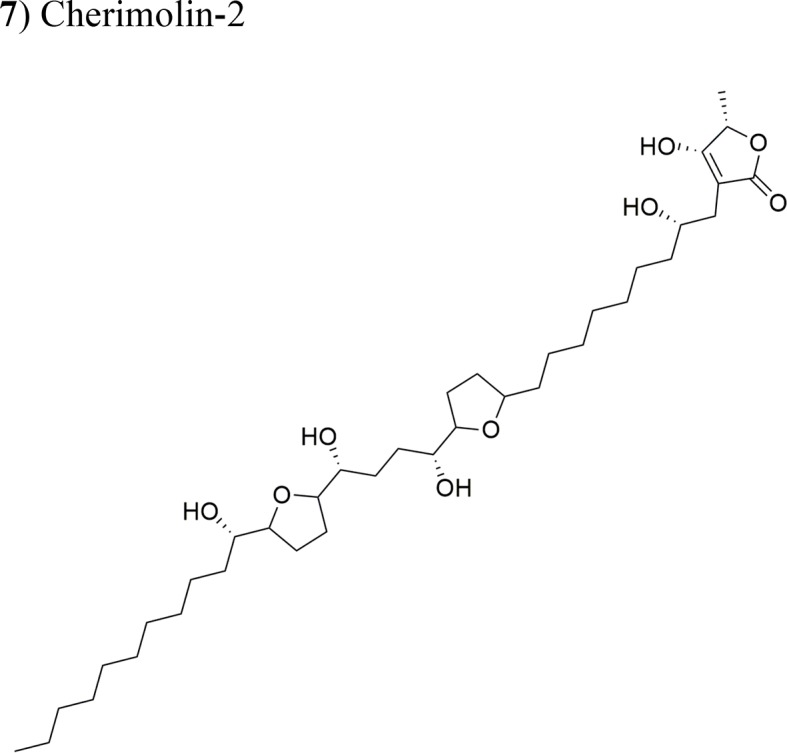			
*Annona montana* Macfad	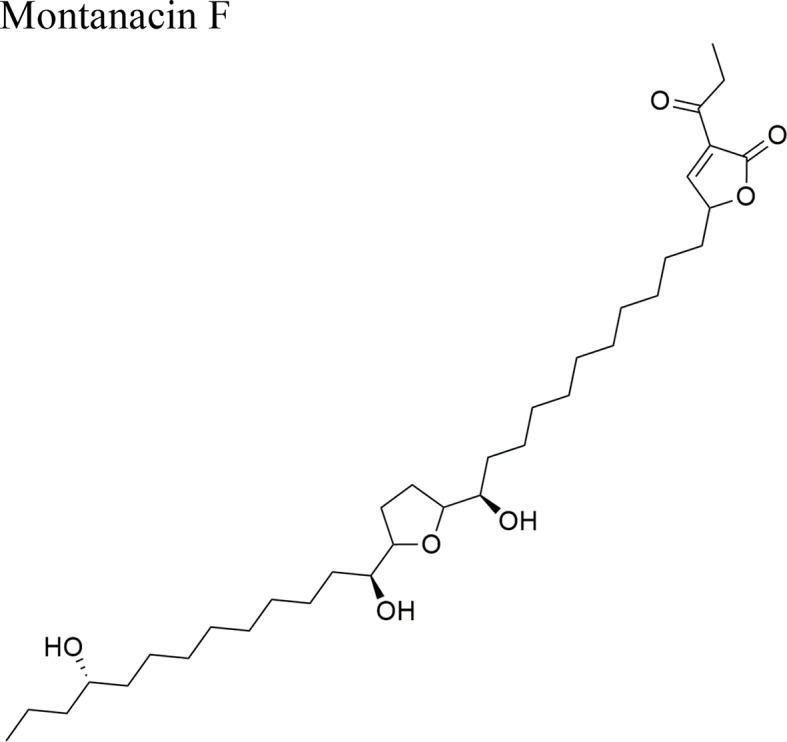	ED_50_ = 0.083 µg/ml/LLC	Terminal lactone unit α-acetonyl-α, β-unsaturated γ-lactone; three OH groups; mono-THF ring	Wang et al., 2002
*Annona muricata* L.	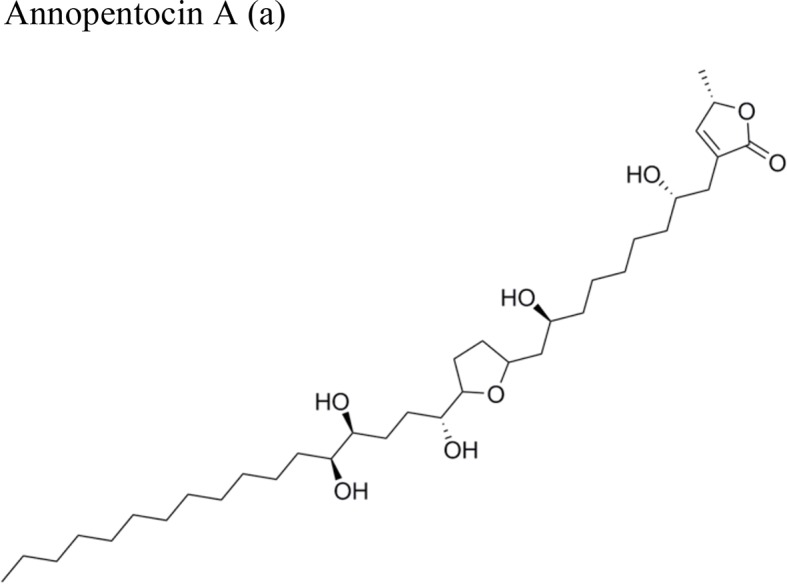	Annopentocin A: ED_50_ (µg/ml) = 0.17/A-549, 1.63/HT-29, 0.6/A-498, 1.14/PC-3, 0.03/PACA-2	Compounds a, b, and c: penta hydroxylated mono-THF ring with one flanking OH on the side chain (hydrocarbon), and an OH on the lactone side	Zeng et al., 1996; Elisya et al., 2014; Moghadamtousi et al., 2015b
	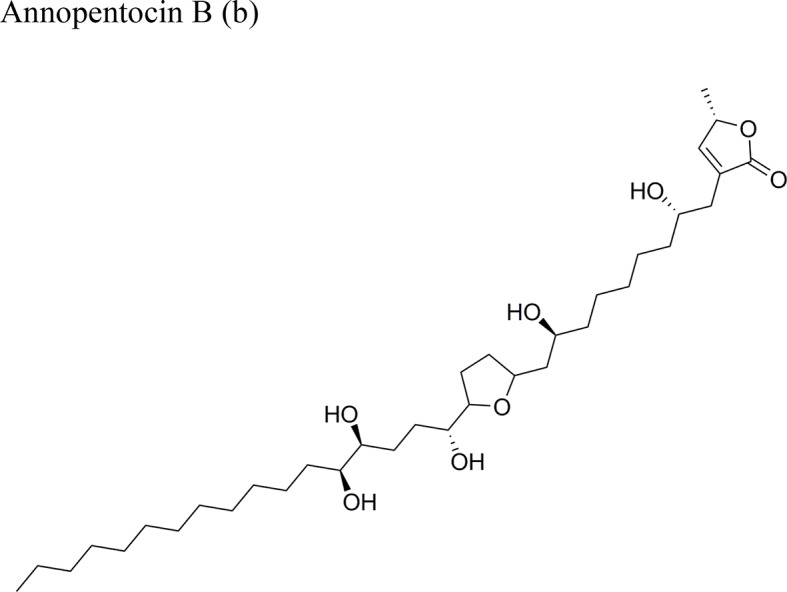	Annopentocin B: ED_50_ (µg/ml) = 0.02/A-549, 3.56/MCF-7, 1.64/HT-29, 0.38/A-498, 0.21/PC-3, 0.16/PACA-2		
	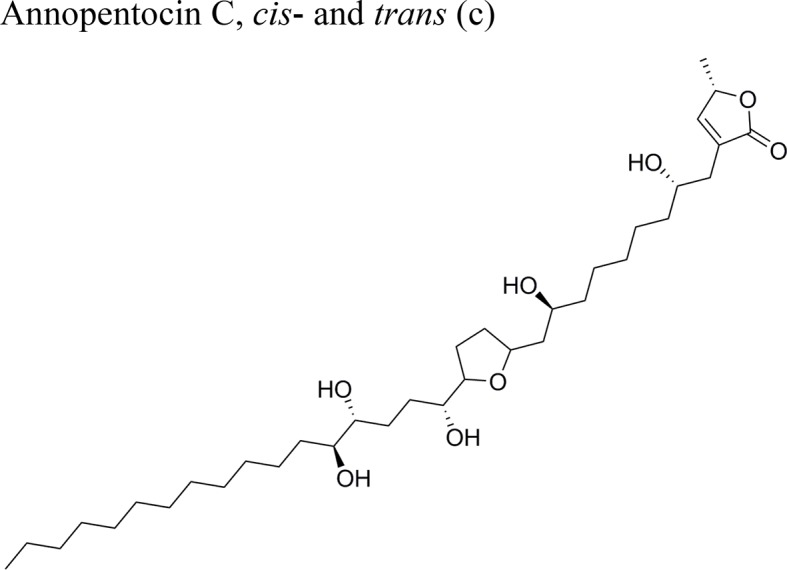	Annopentocin C: ED_50_ (µg/ml) = 0.02/A549, 2.97/MCF-7, 1.24/HT-29, 0.26/A-498, 0.22/PC-3, 0.43/PACA-2		
	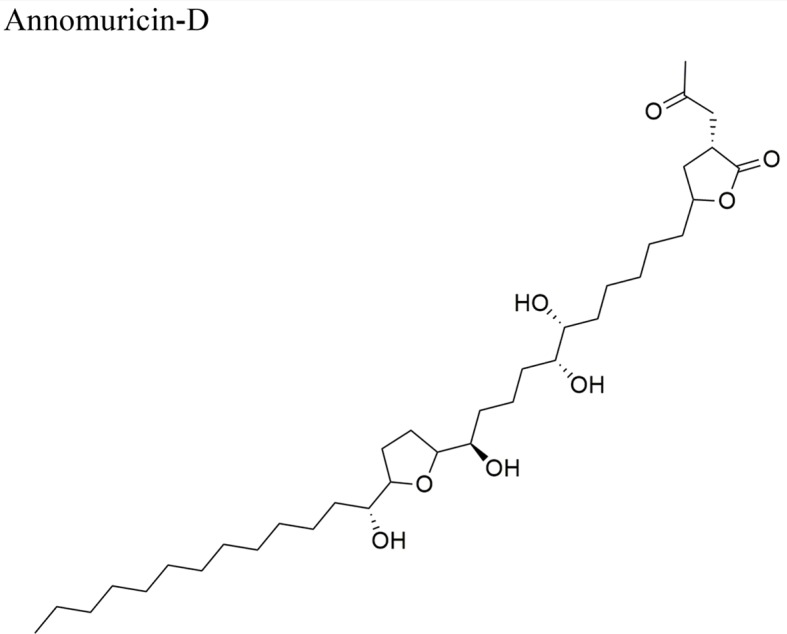	Annomuricin D: ED_50_ (µg/ml) = < 0.01/A-549, 0.6/MCF-7, < 0.01/HT-29, 0.1/A-498, 1.32/PC-3, < 0.916/PACA-2		
	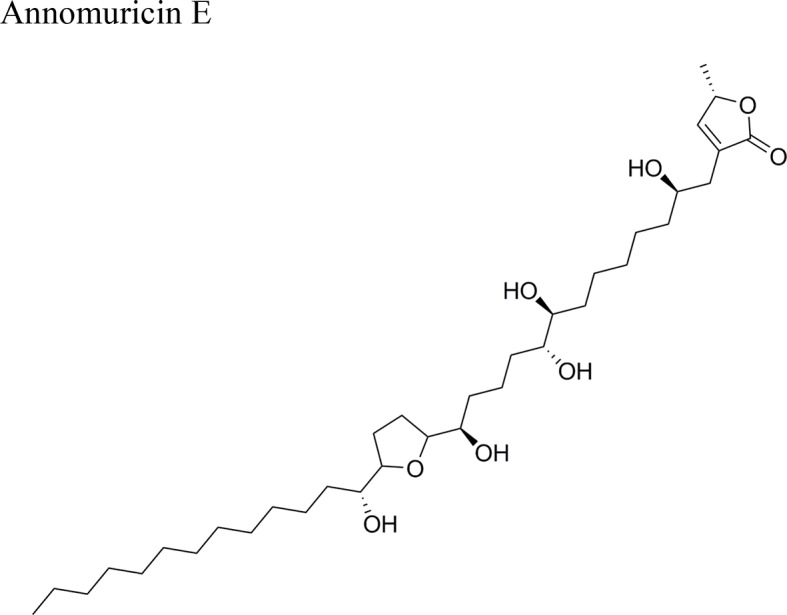	IC_50_ = 1.62 + 0.24 µg/ml/HT-29	Mono-THF bearing two flanking OH and an erythro-diol between the THF and the ketolactone rings	Moghadamtousi et al., 2015b
*Annona squamosa* L.	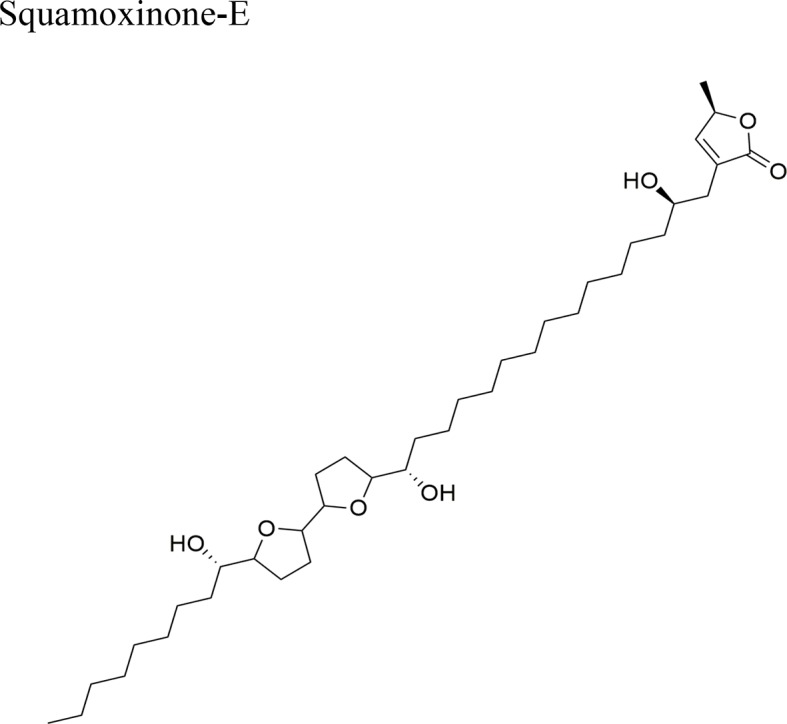	IC_50_ (µg/ml) = 0.103/H460; 0.687/BGC803; 4.19/BEL7402; 0.43/HepG2; 6.56/SMMC-7721	Bis-THF and a 4-OH structure	Miao et al., 2016a
	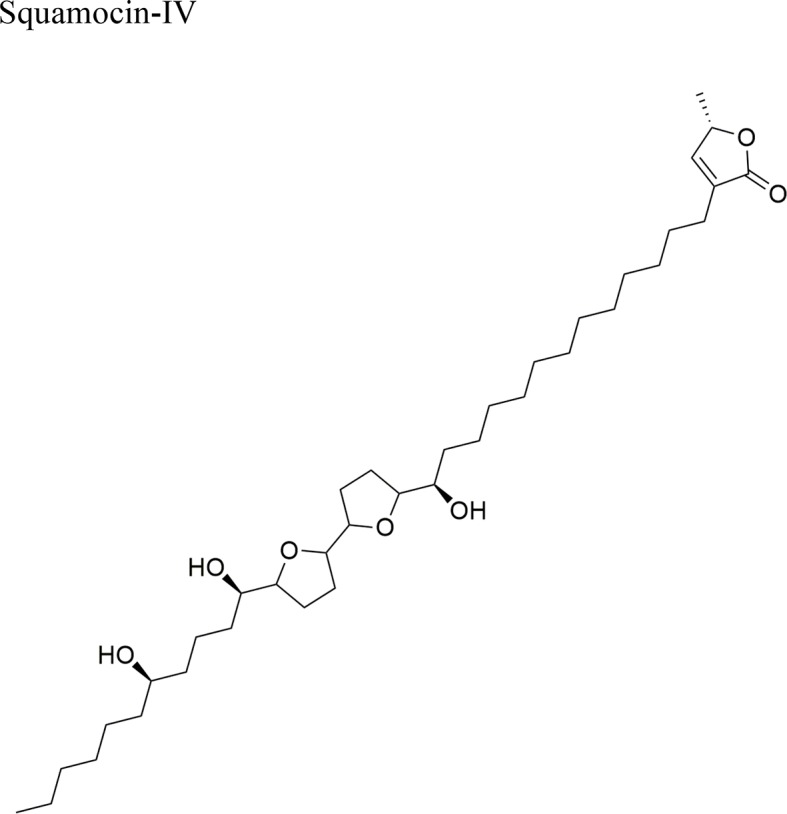	Squamocin-IV: IC_50_ = 0.049 µg/ml/H460 12,15-cis-squamostatin-A: IC_50_ = 17.40 ± 2.09 µg/ml/A-549/taxol (resistant cell line) Squamostatin-D IC_50_ = 16.19 ± 1.98 µg/ml/A-549/taxol (resistant cell line)	Squamocin-IV presence of an ana, β-unsaturated γ-lactone; adjacent bis-THF ring system with two flanking hydroxy groups and four OH groups, one at C-7 Functional groups in 12,15-cis-squamostatin-A and squamostatin-D: non-adjacent bis-THF and fewer hydroxy groups	
	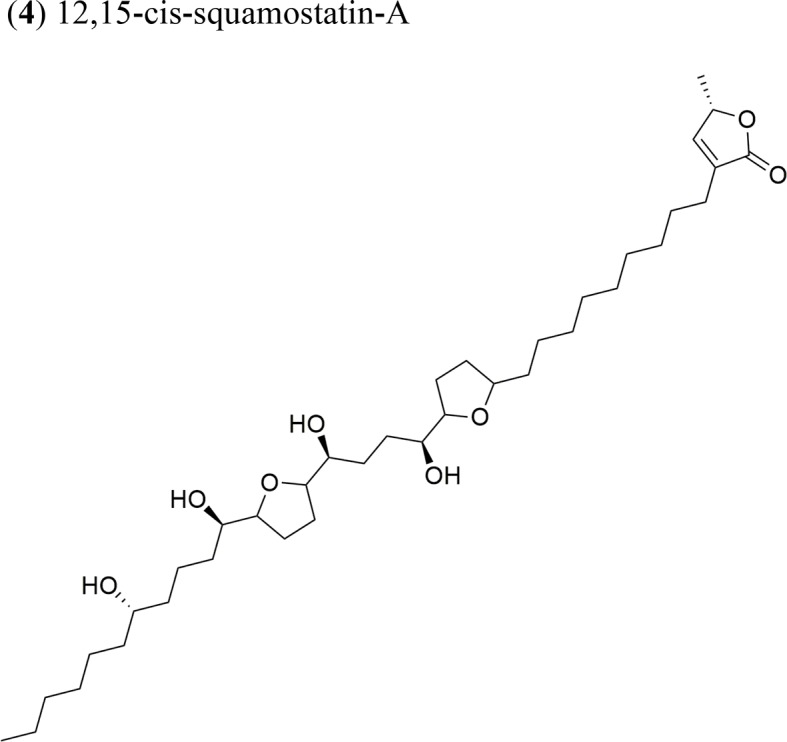			
	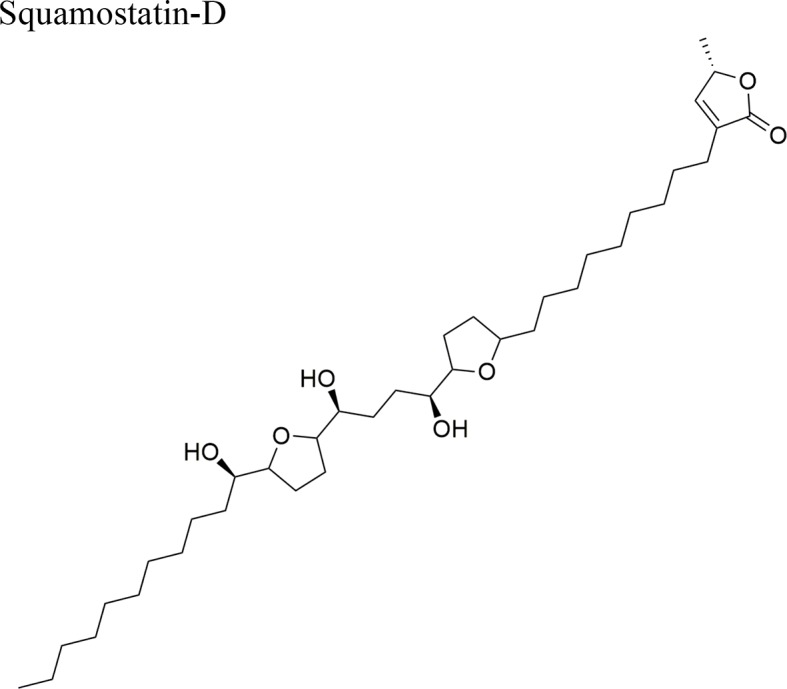			
	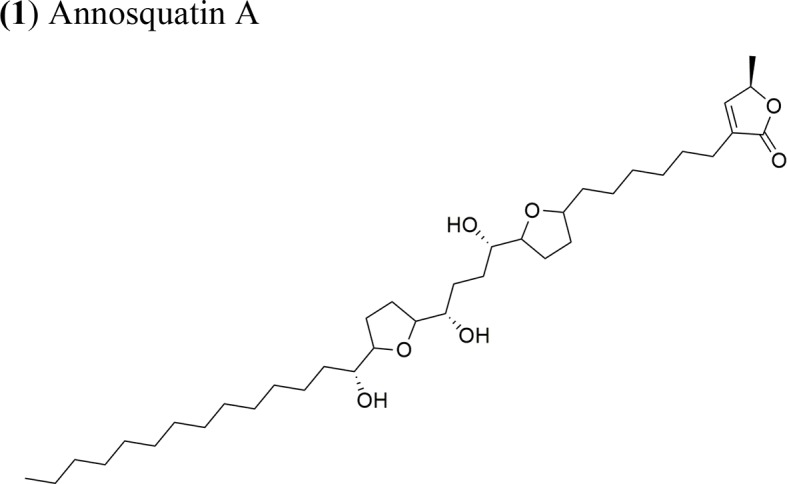	Annosquatin A: IC_50_ = 6.3 × 10^−2^ µg/ml/MCF-7 Annosquatin B: IC_50_ = 8.4 × 10^−2^ µg/ml/A-549	The activity of compounds A and B is related to the three hydroxy groups, two flanking the THF rings and another located in the long hydrocarbon chain gives the cytotoxicity	Chen et al., 2012c
	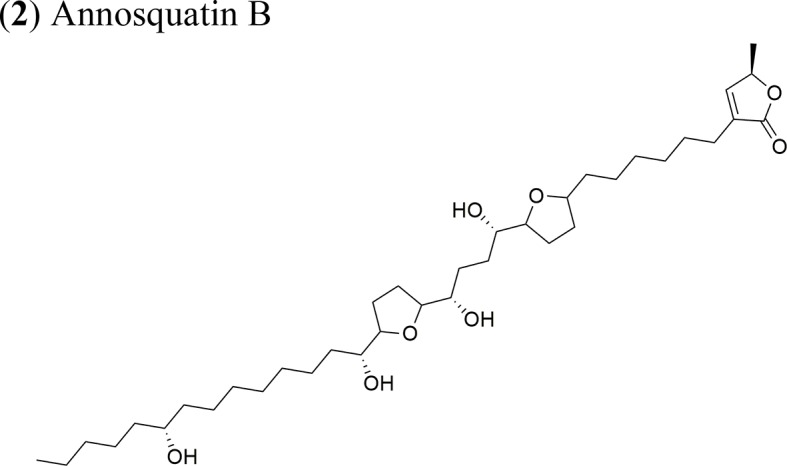			
*Goniothalamus undulatus* Ridl.	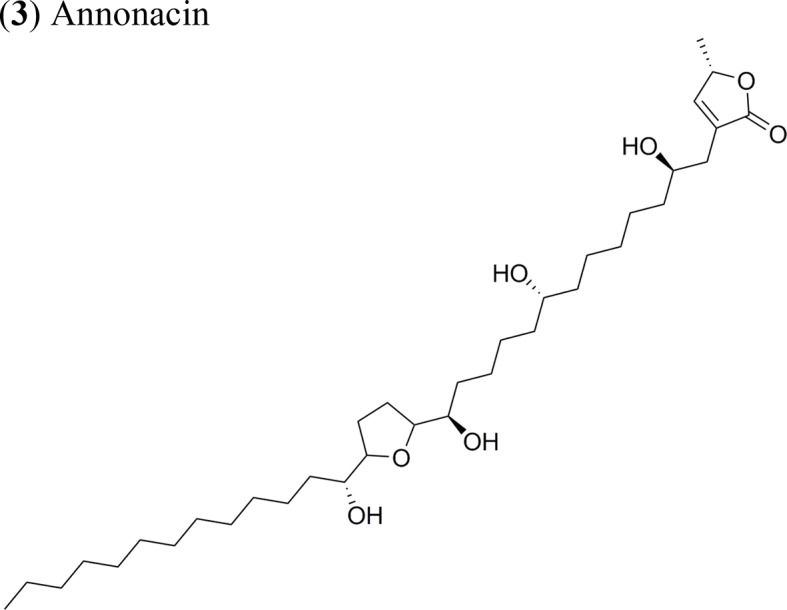	Annonacin: IC_50_ = 0.56 ± 0.02 µM/COR-L23 Goniothalamicin IC_50_ = 1.68 ± 0.09 µM/COR-L23 *Cis*-Goniothalamicin IC_50_ = 1.71 ± 0.27 µM/COR-L23	Functional groups annonacin: mono-THF ring with two flanking hydroxy groups	Tantithanaporn et al., 2011; Yuan et al., 2003
	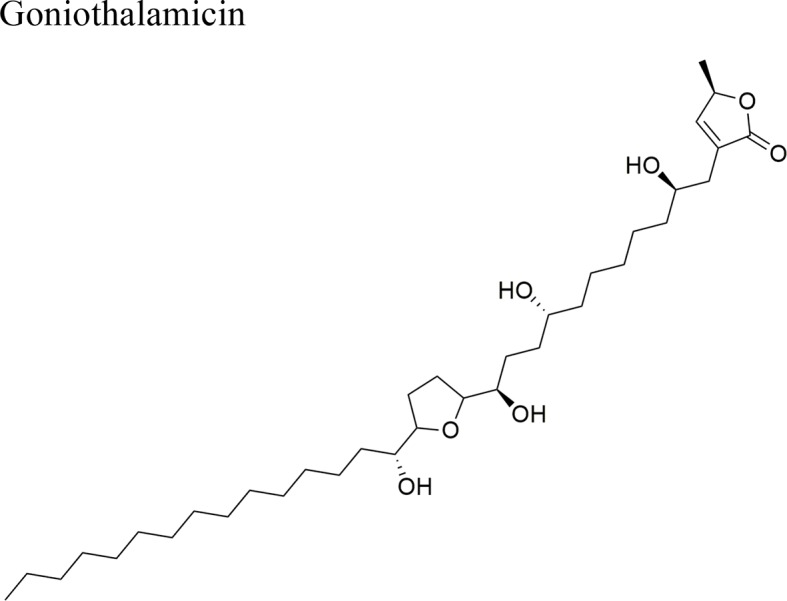			
	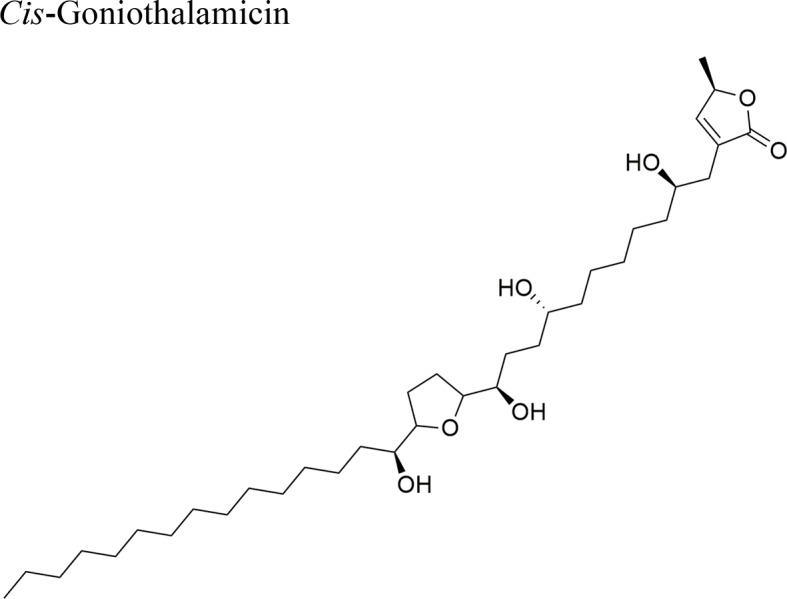			

A-498 (human kidney carcinoma); A-549 (human lung carcinoma); A-549/taxol (lung carcinoma multidrug resistant to taxol); BEL7402 (human hepatocellular carcinoma); BGC803 (human gastric carcinoma); COR-L23 (human lung cancer); H460 (human lung carcinoma); HepG2 (human hepatocellular carcinoma); HT-29 (human colon adenocarcinoma); HCT-15 (adherent colon carcinoma); LLC (Lewis lung carcinoma mouse); MCF-7(human breast carcinoma), PACA-2 (human pancreatic carcinoma); PC-3 (human prostate adenocarcinoma); SMMC-7721 (human hepatocellular carcinoma); SW480 (colon adenocarcinoma)

**Table 2:** Antitumor activity *in vivo* of acetogenins and selected extracts rich in acetogenins.

Acetogenin	Animal model	Antitumor activity	Reference
(**3**) Annonacin	Xenotrasplant MCF-7 tumor cells in nu/nu mice	Tumor growth-inhibitory effect Inhibition of the expression of the proteins ERα, cyclin D1 and Bcl-2	Ko et al., 2011
(**5**) Bullatacin	Xenotrasplant S180 and HepS mice tumor cells in nu/nu mice	Tumor growth-inhibition 65.8% (S180) and 63.4% (HepS) cancer cells. Antitumor activity related to an to the adjacent bis-THF and three hydroxy groups	Chen et al., 2012a
(**6**) Laherradurin	Xenotrasplant HeLa and SW480 tumor cells in nu/nu mice, 7.5 mg/kg/day	Tumor growth-inhibition 64% (HeLa) and 60% (SW480) cancer cells. Antitumor activity related to the adjacent bis THF with β-hydroxy γ-methyl γ-lactone	Schlie-Guzmán et al., 2009
(**7**) Cherimolin-2	Xenotrasplant HeLa tumor cells in nu/nu mice, 25 mg/kg/day Xenotrasplant SW-480 Tumor cells in nu/nu mice 5 mg/kg/day	Tumor growth-inhibition 43% (Hela) cancer cells No significant tumor inhibition in SW-480 xenograft. Antitumor activity related to the non-adjacent bis THF γ-lactone	Schlie-Guzmán et al., 2009
EEAML	AOM-induced colorectal cancer in male rats	Reduction of the colonic aberrant crypt foci (ACF) *in vivo*. Down-regulation of PCNA and Bcl-2 proteins, and up-regulation of Bax in colon. Activity attributed to annomuricin E due to its apoptosis effect by activating the expression of caspase 3, 7 and 9, and upregulation of Bax and downregulation of Bcl-2	Moghadamtousi et al., 2015b
*Annona muricata* L. (graviola capsules Raintree)	Orthotopic tumor xenografts of pancreatic tumor cells (CD18/HPAF) in nu/nu mice Dosis: 50 mg/kg and 100 mg/kg, aqueous extract	Tumor growth-inhibition of 5.8% treated with 50 mg/kg and 50.3% treated with 100 mg/kg of graviola extract The extract reduced pancreatic cells viability through increment of intracellular ROS and necrosis cell death, and cell cycle arrest Diminished MMP3 levels in tumors reducing metastasis and invasion	Torres et al., 2012

AOM, azoxymethane; CD18/HPAF, pancreatic cancer cells; EEAML, ethyl acetate extract of A. muricata leaves; HepS, mouse hepatitis virus; S180, murine sarcoma cells; SW480 (colon adenocarcinoma); ROS, reactive oxygen species; MMP3, matrix metallopeptidase 3.

Apoptosis is a natural strategy of cell death that kills unnecessary or damaged cells. The main genes involved in this process are *p53* and the *bcl2* family; the former is a tumor suppressor, while the later could be either proapoptotic (BAD, BAX and BAK among others) or antiapoptotic (bcl2, and bcl-x) (Okada and Mak, 2004). The primary function of *p53* is to prevent the replication of cells with DNA damage. Therefore, *p53* is inactive, and the damaged cells continue to grow and replicate DNA mutations, resulting in diseases such as cancer (Igney and Krammer, 2002; Okada and Mak, 2004). Thus, research carried out to date can be classed into two main topics: apoptosis induction or apoptosis resistance mechanisms.

Current apoptosis-inducing chemotherapies cause severe secondary effects on patients. As such, the search for less toxic drugs is a priority and natural products are expected to aid in the development of drugs modulating apoptosis. In this context, AAs up-regulate the activity of caspase 3 and 8 (apoptosis effectors), while downregulating the expression of survivin and Bcl-2, thereby enhancing apoptosis. The AA annonacin (**3**) promotes apoptosis in cancer cells by activating the caspase 3 and Bax pathways (Yuan et al., 2003), while squamocin induces apoptosis through the expression of the proapoptotic genes Bax and Bad, which results in the cleavage of PARP and the enhanced activity of caspase 3 in bladder T24 cancer cells (Yuan et al., 2006). This contrasts with previous results where squamocin did not induce apoptosis in breast cancer cells but inhibited proliferation by blocking the cell cycle in the G1 phase (Raynaud et al., 1999).

The methanol extract of *A. reticulata* inhibits the expression of caspases 6 and 9 in colon and liver cancer cells (Mondal et al., 2007), while the organic and aqueous extracts of *A. squamosa* down-regulate the expression of Bcl-2 in MCF-7 breast cancer cells and K-562 leukemia cells, indicating their effect as apoptosis inducers (Pardhasaradhi et al., 2005). In addition, the leaf extract of *A. muricata* induces the expression of caspases 3 and 9 and inhibits cell proliferation by reducing phospho-ERK and phospho-AKT in MIA PaCa-2 cells (Yiallouris et al., 2018).

The AAs also lead to cycle arrest, which has implications for the proliferation of tumor cells. AAs regulate the cell cycle in the G1/S transition by inhibiting cyclin D1 expression in human hepatocellular carcinoma cells (Qian et al., 2015). In this context, the *A. muricata* extract arrests the cell cycle at the G1 phase and decreases the number of cells in the S phase in a concentration-dependent manner by reducing the expression of cyclin D1, an important regulatory protein of the cell cycle (Torres et al., 2012). A similar result was observed for squamocin, which arrests cells in the G1 phase in T24 bladder cancer cells (Yuan et al., 2006). Despite the relevance of the cell cycle, few studies have addressed how AAs affect this mechanism.

Aerobic glycolysis, a mechanism used by tumor cells to obtain energy in the absence of oxygen (Figueroa-González et al., 2016; García-Castillo et al., 2017), is also a target of AAs. Various proteins and glycolytic enzymes are upregulated by HIF-1, an important transcription factor involved in tumor aerobic glycolysis, in cancer cells. Interestingly, the *A. muricata* extract lowered the expression of HIF-1α and NF-κB and the levels of the glucose transporter GLUT1 and the HKII and LDHA enzymes in pancreatic cancer cells (Torres et al., 2012). In addition, the leaf extract of *A. muricata* showed antiproliferative effects in cancer cell lines and promoted cell death by the inhibition of the NKA and SERCA pumps (Yiallouris et al., 2018).

On the other hand, the MDR1 gene codifies the cell membrane glycoprotein P, a key transporter protein that extrudes anticancer drugs from the inside of cells, thereby limiting their intracellular accumulation and diminishing their toxicity (Figueroa-González et al., 2012). Secondary metabolites, such as flavonols, ginsenosides, polyphenols, alkaloids, and resin glycosides, have shown interesting results in modulating P-gp in cancer cell lines (Phang et al., 1993; Beck et al., 1988; Silva et al., 2001; Jodoin et al., 2002; Zhou et al., 2004; Figueroa-González et al., 2012). Furthermore, AAs can down-regulate the expression of the MDR1 and MRP1 genes in drug-resistant human hepatocellular carcinoma, as well as the expression of topoisomerase IIα and glutathione S-transferaseΠ (Qian et al., 2015).

Recently, autophagy had generated interest as a mechanism of cell death. Autophagy is a catabolic process that eukaryotic cells activate when under stress, such as cell starvation or the presence of pathogens (He and Klionsky, 2009). During this process, the cell recycles proteins or non-functional organelles in a multi-step process involving lysosomal degradation to ultimately recover cellular homeostasis (Kenific and Debnath, 2015). As such, the deregulation of the autophagy flux could lead to cancer, as recycling macromolecules, non-functional organelles, and proteins provide cancer cells with high metabolic requirements for cell proliferation (Kenific and Debnath, 2015; García-Castillo et al., 2017). In this scenario, autophagy has become an attractive therapeutic target, and AAs might be capable of inhibiting this process. Liu et al. (2012) reported that the compound AA005 (an acetogenin analog) inhibited ATP production, activated AMPK, and blocked the mTOR Complex 1 pathway to finally induce autophagy in colon cancer cells, and to arrest the cell cycle at the G1 phase. This compound is a mimic of the AAs, where an ethylene glycol ether unit replaces the two THF rings. Such chemical modality confers a superior biological activity, an example of the plasticity of AAs to consider as an inspiration to create new and more powerful molecules with different pharmacological targets.

### Antitumor Activity of Acetogenins in Animal Models

The data regarding the anticancer activity of AAs and the *Annona* extracts are particularly promising since these compounds exhibited antitumor activity in animal models. Studies *in vivo* provide key information about the performance of the drug in an entire organism and allow evaluating not only their antitumor effect but also their toxicity in different organs. Importantly, the animals are experimental subjects that enable to perform clinical follow-up, including clinical outcome assessment, disease-free progression and survival, and disease recurrence.

The ethyl acetate extract of *A. squamosa* that is rich in the AAs 12,15-*cis*-squamostatin-A (**4**) and bullatacin (**5**) reduced the tumor growth of hepatocellular tumors in mice at a maximum inhibitory rate of 69.55% compared to the positive control (cyclophosphamide) (Chen et al., 2012b). It was suggested that the *cis* configuration in one of the components could be responsible for the cytotoxic activity of the herbal preparation. Moreover, bullatacin (**5**) isolated from *A. squamosa* at a dose of 15 μg/kg effectively reduced tumor growth in mice bearing S180 and HepS xenografts by 65.8 and 63.4%, respectively. These results are superior to those obtained using higher concentrations of taxol (40 μg/kg). Bullatacin (**5**) possesses an adjacent bis-THF moiety and three hydroxy groups, which likely constitute the bioactive structure (Chen et al., 2012a).

The seed oil of *A. squamosa* inhibited 53.54% of tumor growth in mice bearing H22 cells (Chen et al., 2016) and reduced the expression of IL-6, Jak, and various phosphorylated signal transducers and activators of the transcription p-Stat3 pathway. It was also reported that the α, β-unsaturated γ-lactone moieties present in AAs are Michael reaction acceptors (Ji et al., 2012), which inhibit Stat3 activation, a therapeutic target involved in cell proliferation, apoptosis, inflammation, and angiogenesis (Cafferkey and Chau, 2016). The extract of *A. muricata* slowed tumor growth in pancreas xenografts (Yiallouris et al., 2018), reducing metastasis by diminishing the levels of the metalloproteinase-9, and promoting cancer cell death through necrosis (Torres et al., 2012). In a prostate xenograft, the *A. muricata* extract enriched with flavonoids improved the bioavailability and showed lower toxicity than the extract enriched with AAs (Yang et al., 2015).

Laherradurin (**6**) and cherimolin-2 (**7**) were isolated from the medicinal plant *A. diversifolia* and tested *in vivo* against cervical and colorectal cancer cells (Schlie-Guzmán et al., 2009). Both AAs reduced the size of HeLa tumors with similar values to those of doxorubicin; both compounds also exhibited antiproliferative activity *in vitro* against the same cancer cell line. These results are in agreement with other reports, where the most active molecule was reported to be laherradurin (**6**), which possesses adjacent bis-THF moieties and a β-hydroxy γ-methyl γ-lactone structure, while cherimolin-2 (**7**) that contains a non-adjacent bis-THF moiety and a γ-lactone unit exhibits a reduced antitumor activity. **Table 2** outlines the antitumor activities of various AAs in animal models.

### Toxicity Studies

Experiments with animals should include acute oral toxicity protocols to test chemicals and observe signs of toxicity; these minimize the number of animals required, assure a correct dose administration in the experiments, and avoid suffering (OECD, 2008). However, to date, there is little information regarding the toxicity of AAs or *Annona* extracts. For instance, the LD_50_ for the ethanol extract of *A. muricata* in mice was 1.67 g/kg according to Sousa et al. (2010). Arthur et al. (2011) reported the LD_50_ (<5 g/kg) of the aqueous extract, recording that higher dose might damage the kidneys. Also, it was observed that the seed extract of *A. squamosa* could cause liver damage (Miao et al., 2016b), and the *A. muricata* extract enriched with flavonoids exhibited reduced toxicity in a prostate xenograft (Yang et al., 2015). Cherimolin-2 isolated from *A. diversifolia* exhibited toxic effects and death in SW480 xenografts in doses over 5 mg/kg/day (Schlie-Guzmán et al., 2009).

As explained before, AAs can inhibit the complex 1 of the respiratory chain through the electron chain transport in the mitochondria. The AAs join and block the NADH enzyme, which is usually overexpressed in cancer cells, inhibiting ATP production, eventually leading to cell death. Presumably, such mechanism suggests that the AAs are “harmless” to normal cells; still, more studies should be performed to assure the selectivity of these molecules.

## Conclusion

Chemotherapy is not specific to cancer cells; it causes several undesirable side effects like damage to normal tissues and organs. However, the most important aspect of conventional chemotherapy is that in a significant number of cases, cancer cells develop resistance mechanisms that enable tumor progression and metastasis.

The search for new drugs in nature is not new. Several derivatives of natural substances are currently used to treat different diseases. For instance, taxol (paclitaxel) that was discovered by the traditional knowledge is considered the most profitable cancer treatment in the market. Interestingly, paclitaxel is an antimitotic drug inhibiting cell proliferation in cell cultures but, in tumors, has been documented to induce multipolar divisions, which depicts how a molecule can have different mechanisms of action depending on *in vitro* or patient conditions. This alkaloid has 30 years of history and illustrates the main obstacles of working with natural products, their limited biologic availability and the costs of production, and also the incredible efficacy killing cancer cells.

Acetogenins are versatile anticancer molecules causing tumor cell death by different mechanisms. They can modulate the exclusion of chemotherapeutics drugs out of cancer cells and are strong apoptosis inducers. Their bioactive flexibility is reflected in their ability to regulate the cell cycle by arresting cells in phase G1, promoting apoptosis by the inhibition of various proteins, and even induce autophagy. Moreover, their metabolic interactions, specifically related to the transcription factors HIF1 and STAT3 and its repercussions in energy consumption, angiogenesis, inflammation, and metastasis, stand out. The antitumor activity of AAs *in vivo* is promising (bullatacin, laherradurin, and cherimolin-2 are examples). However, the preclinical data are not sufficient to obtain a good understanding of the pharmacodynamics and kinetics of AAs, and more acute toxicity and solubility tests are needed to assure safety and the possibility of clinical trials with humans. Additionally, the incorporation of different ligands (i.e., antibodies, vitamins, and peptides) or the preparation of tumor-specific derivatives could improve AA activity and yield more suitable drugs.

Plant extracts are chemically complex, and their curative properties often depend on the interactions among compounds and their proportions within the extract. There is still much to discover regarding the impacts of AAs in cancer. This field provides the opportunity of finding new molecules for the treatment of this complex disease. Standardizing extracts is, therefore, a possible alternative to using herbal supplements, especially in plants where the pharmacological activities are based on the combination of more than one compound. This mini-review lists some previously studied AAs that present antitumor activity and could have a future in clinical cancer research.

## Author Contributions

NJ-H contributed to conception, writing, and discussion of the article; CP-P contributed substantially to discussion and revision for the manuscript; VC-T prepared all figures and tables; MM-V and AG-E contributed to the chemical section of the article; AZ-D contributed significantly in the molecular biology discussion of the article. All the authors discussed, revised, and approved the final version of the manuscript to be published.

## Funding

This work was supported by the Consejo Nacional de Ciencia y Tecnología (CONACYT), Mexico (grant number 285884). VC-T was funded by CONACYT (PhD grant number 267787).

## Conflict of Interest Statement

The authors declare that the research was conducted in the absence of any commercial or financial relationships that could be construed as a potential conflict of interest.
